# Complete Genome Sequence of a Canadian Klebsiella michiganensis Strain, Obtained Using Oxford Nanopore Technologies Sequencing

**DOI:** 10.1128/MRA.00960-20

**Published:** 2020-11-12

**Authors:** Mingsong Kang, John Chmara, Marc-Olivier Duceppe, Beverley Phipps-Todd, Hongsheng Huang

**Affiliations:** a Canadian Food Inspection Agency, Ottawa Laboratory-Fallowfield, Ottawa, Ontario, Canada; Broad Institute

## Abstract

Klebsiella michiganensis is a Gram-negative opportunistic pathogen that is associated with many hospital-acquired infections in humans. Here, we report the complete genome sequence of a K. michiganensis strain isolated from a Canadian wastewater treatment facility.

## ANNOUNCEMENT

The genus *Klebsiella* is in the *Enterobacteriaceae* family, with 22 confirmed Gram-negative coliform bacterial species (https://lpsn.dsmz.de/genus/klebsiella) that are found in various environmental habitats, including soil, food, plants, insects, and water, and have been associated with nosocomially acquired diseases in humans (1, 2). Klebsiella michiganensis was first reported in 2013 and was found to be associated with infections in humans and animals (3–6).

This article reports the complete genome sequence of a K. michiganensis strain (biosolid 27) that was isolated from a pretreated sample from a Canadian wastewater treatment facility. The strain was isolated using a procedure to test Escherichia coli (7) by preenrichment for coliforms in lauryl sulfate tryptose broth, selective enrichment in Escherichia coli broth, isolation using Levine's eosin methylene blue agar, and biochemical identification. The isolate was identified as K. michiganensis based on genome sequences in this study.

The isolate was kept at −80°C in tryptic soy broth (TSB) containing 25% glycerol before sequencing. Genomic DNA was extracted and purified from overnight cultures grown in TSB at 37°C using the Nanobind CBB Big DNA kit (cells, bacteria, blood) followed by the Short Read Eliminator XS kit (Circulomics, Inc., Baltimore, MD, USA) and was quantified using a Qubit v3.0 fluorometer (Life Technologies, Thermo Fisher Scientific, Inc., Ottawa, Canada). Libraries for MinION sequencing were prepared without shearing using the 1D ligation sequencing kit (SQK-LSK108), and DNA was barcoded with the native barcoding expansion kit (EXP-NBD103) (Oxford Nanopore Technologies, Oxford, UK). The final library was run on a FLO-MIN106 (R9.4.1) flow cell for 48 h on the MinION platform (Oxford Nanopore Technologies). Resulting fast5 reads were base called using the high-accuracy base-calling algorithm in Guppy v3.4.5. MinION reads (fastq files) were trimmed by Porechop v0.2.3 with default parameters and filtered by Filtlong v0.2.0 using the parameters “keep percent 90” and “target bases 700000000.”

MinION reads were assembled with Shasta v0.4.0 using the parameters “MinHash.minBucketSize 5,” “MinHash.maxBucketSize 30,” “MinHash.minFrequency 5,” “Align.minAlignedFraction 0.4,” “Assembly.consensusCaller Bayesian:guppy-3.0.5-a,” “Reads.minReadLength 5000,” “memoryMode filesystem,” and “memoryBacking 2M”; the assembled data were polished with Medaka v0.13.0. The sequencing coverage depth was determined using minimap2 v2.17 (8) by mapping the long reads against the assembled genomes, and the results were analyzed using Qualimap v2.2.1 and Tablet v1.19.09.03. The coverage was assessed manually across the entire genome, with the mean coverage determined by SAMtools v1.10 (9). Gene predictions and annotations were performed using NCBI Prokaryotic Genome Annotation Pipeline (PGAP) (10). Prophage sequences were analyzed using PHASTER (11). Antimicrobial resistance (AMR) genes were identified by ABRicate v1.0.1.

The genome of the strain contains a single chromosome. The assembled genome was submitted to GenBank. The total length, protein count (number of coding sequences [CDSs]), GC content, and numbers of tRNAs, prophages, and AMR genes of the genome assembly are provided in Table 1. The median total length, number of CDSs, and GC content of K. michiganensis genome assemblies available in GenBank are similar to those of the K. michiganensis strain (Table 1).

**TABLE 1:** Genomic characteristics of a Klebsiella michiganensis strain isolated from a Canadian wastewater treatment facility

Strain or sequence	No. of contigs	Total length	Coverage (×)	GC content (%)	No. of CDSs	No. of predicted genes	AMR genes	No. of tRNAs	No. of prophages
Biosolid 27	1	5,891,206 bp	60.4	56.1	5,374	5,490	*oqxB9*, *oqxA10*, *bla*_OXY-1_, *fosA*	85	3
GenBank sequences (median)^*a*^		6.1921 Mbp		55.8	5,690				

a A total of 204 genome assemblies were identified on 16 September 2020.

### Data availability.

This whole-genome shotgun project has been deposited in DDBJ/ENA/GenBank under the accession number CP054159. The version described in this paper is version 1. Oxford Nanopore Technologies base-called fastq files are available in the NCBI Sequence Read Archive (SRA) under the accession number SRR12464721.
